# Identification and Characterisation of *Pseudomonas* 16S Ribosomal DNA from Ileal Biopsies of Children with Crohn's Disease

**DOI:** 10.1371/journal.pone.0003578

**Published:** 2008-10-31

**Authors:** Josef Wagner, Kirsty Short, Anthony G. Catto-Smith, Don J. S. Cameron, Ruth F. Bishop, Carl D. Kirkwood

**Affiliations:** 1 Enteric Virus Group, Murdoch Childrens Research Institute, Department of Paediatrics, Royal Children's Hospital, Parkville, Victoria, Australia; 2 Department of Microbiology & Immunology, University of Melbourne, Melbourne, Victoria, Australia; 3 Department of Gastroenterology & Clinical Nutrition, Royal Children's Hospital, Parkville, Victoria, Australia; German Cochrane Center, Germany

## Abstract

Molecular analysis of bacterial 16S rRNA genes has made a significant contribution to the identification and characterisation of bacterial flora in the human gut. In particular, this methodology has helped characterise bacterial families implicated in the aetiology of inflammatory bowel disease (IBD). In this study we have used a genus specific bacterial 16S PCR to investigate the prevalence and diversity of *Pseudomonas* species derived from the ileum of children with Crohn's disease (CD), and from control children with non-inflammatory bowel disease (non-IBD) undergoing their initial endoscopic examination. Fifty eight percent of CD patients (18/32) were positive using the *Pseudomonas* PCR, while significantly fewer children in the non-IBD group, 33% (12/36), were PCR positive for *Pseudomonas* (p<0.05, Fischer's exact test). *Pseudomonas* specific 16S PCR products from 13 CD and 12 non-IBD children were cloned and sequenced. Five hundred and eighty one sequences were generated and used for the comparative analysis of *Pseudomonas* diversity between CD and non-IBD patients. *Pseudomonas* species were less diverse in CD patients compared with non-IBD patients. In particular *P.aeruginosa* was only identified in non-IBD patients.

## Introduction

Crohn's disease (CD) is a chronic inflammatory condition of the gastrointestinal tract, predominantly affecting the ileo-caecal region. CD is increasing in incidence in numerous countries throughout the world [1], [2], [3]. One model of CD suggests that it is initiated in genetically susceptible individuals by an infectious agent capable of triggering a breakdown in the regulatory constraints on the mucosal immune system and leads to an immune mediated tissue injury. However, the infectious agent(s) that trigger these responses is unclear. Several putative susceptibility genes have been implicated in CD, including CARD15, IL-23R and TLR-4 [4], [5], [6]. The role of CARD15 in sensing bacterial peptidoglycan, and the association between mutant forms of this gene and CD, further suggest that micro-organisms may play a key role in disease aetiology.

*Mycobacterium avium* subspecies *paratuberculosis* [7] has been the most widely implicated aetiological agent of CD [8]–[10]. Adherent-Invasive *E.coli* [11], *Listeria monocytogenes* [12], *Pseudomonas* [13], [14] and *Yersinia* [15] have also been proposed as triggers of CD. Some authors have also argued that CD results from a loss of tolerance to the normal flora of the gastrointestinal tract [16]. This hypothesis is supported by observations that CD does not occur in gnotobiotic rats, and that some CD patients have significantly higher antibody levels to commensal gut bacteria [17].

It has also been suggested that CD is the result of dysbiosis i.e. an imbalance between ‘beneficial’ (non-inflammatory) commensals and ‘harmful’ (pro-inflammatory) commensals [18]. Using bacterial 16S ribosomal RNA (rRNA) gene sequencing, the accepted standard of bacterial identification [19], studies have demonstrated a significant difference in the gut flora of CD patients compared with control populations, providing support for the dysbiosis hypothesis [20]–[22]. Such studies report that members of the phylum *Bacteriodetes* and *Proteobacteria* are increased in CD patients. A preliminary study reported by Eckburg and Relman using 16S rDNA PCR also identified significantly more *Proteobacteria* phylum-associated 16S rDNA sequences (preliminary *E. coli* and *Pseudomonas* species) from colonic tissue samples obtained from patients with CD than from non CD patients [23]

Bacteria within the genus *Pseudomonas* have also been associated with the development of CD. Several *Pseudomonas* species are opportunistic pathogens including *P.aeruginosa* and *P.fluorescens* [25], [24]. Some, such as *P.aeruginosa* and *P.putida*, are also minor members of the normal gastrointestinal microbiota [26]–[28]. Early studies reported that variants of *Pseudomonas*-like bacteria were detected in the gastrointestinal tract of CD patients and found to be absent in the gastrointestinal tract of patients without inflammatory bowel disease (IBD) [29]–[31]. More recently, a T-cell superantigen (I2), encoded by *P.fluorescens*, has been associated with lesions in CD patients [13], [14]. However, many of these studies were performed on adult patients with long standing disease, who had experienced a variety of treatment regimes, including antibiotics that would have altered intestinal flora.

A series of preliminary subtractive hybridisation experiments conducted in our laboratory comparing DNA extracted from CD and non-IBD gut biopsies detected *Pseudomonas* specific 16S rDNA in the differentially expressed populations (Kirkwood, unpublished observations). These experiments implied that the role of *Pseudomonas species* in early onset CD should be investigated further.

Here we present a study investigating the role of *Pseudomonas* species in CD and non-IBD paediatric patients presented for initial diagnosis prior to medical treatment. Specifically, the diversity of *Pseudomonas* species in the mucosa of gastrointestinal tracts of children with and without CD was investigated. We have conducted a *Pseudomonas* specific 16S ribosomal analysis on ileal tissue obtained from CD and non-IBD patients, and subsequently cloned and sequenced PCR products from each patient. The resulting *Pseudomonas* gene libraries were compared using LIBSHUFF (LIBrary SHUFFling) analysis [32] and DOTUR (Distance Based Operational Taxonomic Unit and Richness Determination) analysis [33].

## Results

### Identification of *Pseudomonas* in clinical specimens obtained from CD and non-IBD patients

*Pseudomonas* specific 16S (P16S) ribosomal DNA PCR was performed on DNA extracted from 64 ileal biopsy specimens. Fifty eight percent of CD patients (18/32) were *Pseudomonas* positive, compared with 33% (12/36) non-IBD patients. Statistical analysis revealed a significant difference between *Pseudomonas* positive patients in the CD group compared with the non-IBD group (p = <0.05, Fisher's exact test).

### *Pseudomonas 16S* sequences diversity in CD and non-IBD patients

The diversity of *Pseudomonas* species present between the two patient groups was analysed by comparing sequences of the 617 bp P16S PCR products from 13 CD and 12 non-IBD patients. A minimum of 20 sequences were analysed for each PCR product, and were used to create a partial P16S gene library for each patient group. Each library of P16S sequences were aligned using ClustalW. A PHYLIP generated DNA distance matrix was calculated using the Jukes-Cantor method and used for LIBSHUFF. LIBSHUFF analysis showed that the P16S gene libraries of CD and non-IBD control patients were significantly different (p<0.025).

**Figure 1:**
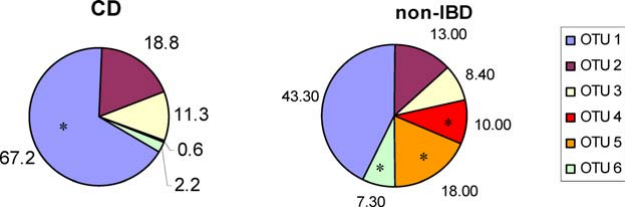
Diversity analysis of *Pseudomonas* 16S ribosomal sequences obtained from Crohn's disease patients and non-inflammatory bowel disease control patients. Five hundred and eighty one sequences from the Crohn's disease (CD) and non-inflammatory bowel disease (non-IBD) patient libraries were grouped into operational taxonomic units (OTU) using a sequence similarity threshold of 97%. The fraction of sequences in each OTU and patient group were calculated and presented. * Significant more sequences in these OTUs compared to the other patient group (p<0.01, Fisher exact test).

All 581 sequences from the P16S gene library were organised into operational taxonomic units (OTU) using DOTUR. Each OTU consists of a group of P16S sequences with 97% sequence similarity. The P16S sequences were grouped into 6 OTUs of which five were identified in the CD clone library and all six in the non-IBD clone library (supporting table S3). Five OTUs were common to both clone libraries. One OTU was unique to the non-IBD clone library. The majority of sequences (67.2% CD and 43.3% non-IBD) were found in the OTU 1. The second most commonly identified sequences were found in OTU 2 (18.8% CD and 13% non-IBD). OTU 3 sequences were present in 11.3% of the CD group and in 8.4% of the non-IBD group. OTU 4 represented 10% of sequences in the non-IBD group whilst this OTU was only a minor fraction within the CD group (0.6%). OTU 5 sequences were only identified in the non-IBD group (18%). OTU 6 was the least common type, identified in 7.3% of non-IBD and 2.2% of CD sequences (Figure 1). The number of sequences in each individual OTU was tested against the number of sequences in the remaining OTUs between the two patient groups using Fischer's exact test. A significant difference was identified in OTU 1 and OTUs 4, 5, and 6 between CD and non-IBD patient groups (p<0.01) (Figure 1 marked with *).

### Phylogenetic analysis of *Pseudomonas* 16S gene library from CD and non-IBD patients

**Figure 2:**
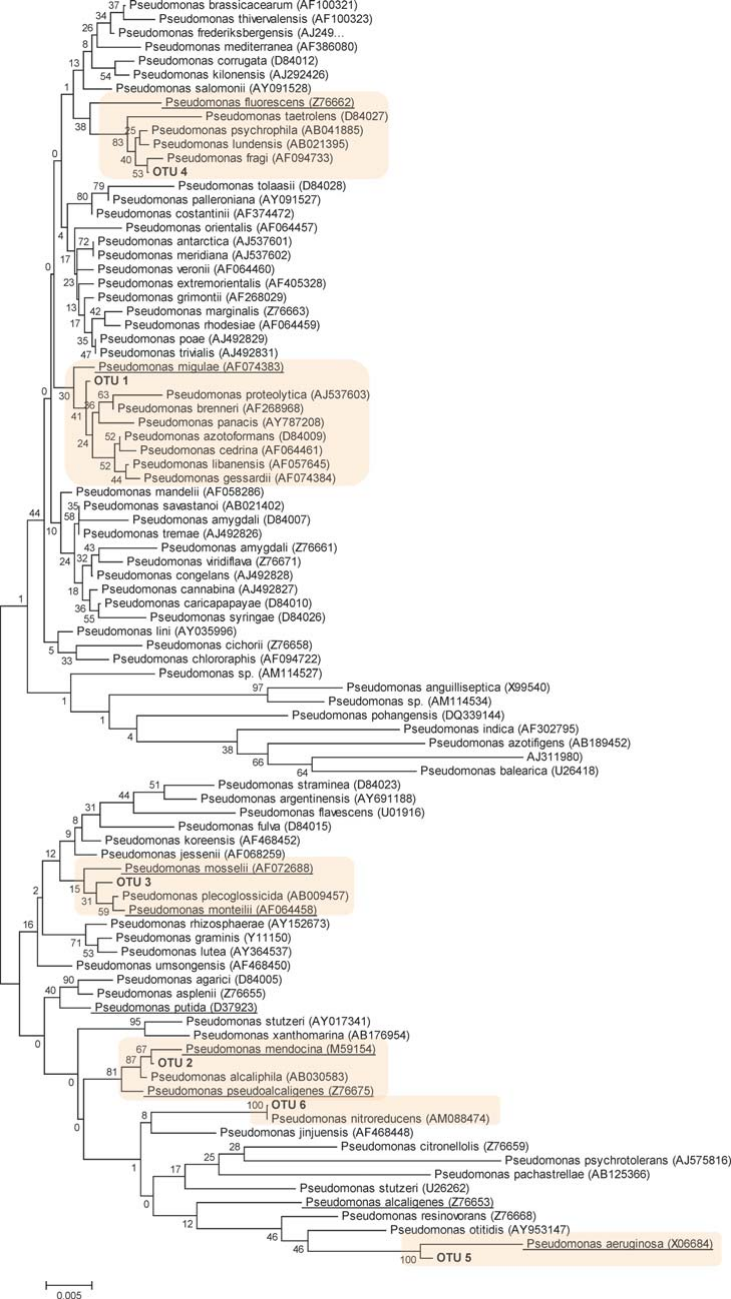
Phylogenetic tree generated from *Pseudomonas* type strains and consensus sequences from OTUs (OTU1-6) at sequence similarity threshold of 97%. Bootstrap values are base on 500 replications. The scale bar represents the nucleotide substitution per site. Underlined reference sequences are known human pathogenic strains. Shadowed box are considered as closely related species within each OTU group.

A consensus sequence of each of the 6 OTUs was generated, representing the 581 sequences determined from *Pseudomonas* 16S PCR products. Figure 2 shows the phylogenetic analysis of each OTU consensus sequences and sequences from 85 *Pseudomonas* reference strains. Each individual OTU clustered into distinct groups with specific *Pseudomonas* reference strains. OTU 1 grouped with *P.migulae*, *P.proteolytica*, *P.brenneri*, and *P.panacis*. OTU 2 grouped with *P.mendocina*, *P.alcaliphila* and *P.pseudoalcaligenes*. OTU 3 grouped *P.mosselii*, *P.plecoglossicida* and *P.monteilii*. OTU 4 grouped with *P.fragi*, *P.lundensis*, and *P.psychrophila*. OTU 5 formed a distinct group with *P.nitroreducens* and OTU 6 formed a distinct *P.aeruginosa* group.

## Discussion

In this study we have conducted a molecular analysis using 16S ribosomal DNA to compare the composition and distribution of *Pseudomonas* species identified in ileal specimens obtained from CD and non-IBD paediatric patients. This study extends on previous studies which reported significant differences in bacterial composition between IBD and non-IBD patients at higher bacterial hierarchy levels [20], [21], 38, 39. In the present study, *Pseudomonas* 16S (P16S) rDNA was detected in a proportion of ileal biopsy samples analysed from both patient groups. This is consistent with previous findings that describe *Pseudomonas* as normal, low level members of the gut microbiota [26], [28]. *Pseudomonas* species were found to be significantly more prevalent in biopsy specimens obtained from CD patients (58%) when compared to specimens from non-IBD patients (33%) (p<0.05, Fisher's exact test). This finding suggests that there is an altered mucosal environment in CD patients that favours the growth of *Pseudomonas* species, which correlates with previous studies that reported *Pseudomonas* like species in CD patients and not in non-IBD patients [29], [30].

The P16S sequence libraries between the two patient groups differed significantly from each other (p<0.025, LIBSHUFF analysis). To analyse the diversity of *Pseudomonas* species within CD and non-IBD libraries, all 581 P16S sequences were grouped into operational taxonomic units (OTUs). The non-IBD gene library was more diverse than the CD library with 6 OTUs, compared to 5 OTUs in the CD library suggesting that the decline in diversity may occur early in onset disease. All patients used in this study were children, with an average age of 11.7 years. Thus, environmental factors (such as smoking and oral contraceptive use) which typically affect studies on adults with CD, are unlikely to have significantly affected the data [40], [41]. These results were not confounded by prolonged treatment with anti-inflammatory drugs or antibiotics since all patients were recruited at initial clinical examination.

Differences in the distribution of *Pseudomonas* species were identified in the CD and non-IBD P16S libraries. The proportion of sequences found in 4 of 6 OTUs were significantly different between the groups (p<0.01, Fischer's exact test). This shifting in numbers of different *Pseudomonas* species between patient groups might contribute to an imbalance between beneficial and harmful microbiota leading to the inflammatory changes seen in CD.

It is widely recognised that *Pseudomonas* is a very diverse group of bacteria, with many species capable of causing human disease. Pathogenic species are contained within each OTU were identified in this study. For example, *P.migulae*, reported to be present in joints in patients with chronic reactive arthritis [42] was within OTU 1. While *P.pseudoalcaligenes*, an organism isolated from respiratory tract culture of cystic fibrosis patients [43] was within OTU2. *P.mosselii* and *P.monteilii* species were closely related to sequences within OTU 3. These novel species were isolated from clinical specimens such as urine, sputum, placenta, skin, and bronchial samples [44]. The pathogenic strain *P.fluorescens* indentified in OTU 4 has been postulated to be implicated in CD [13], [14]. However, our study does not support a role for *P.fluorescens* in CD since there was a significant reduction in species within the OTU 4 group in CD compared with non-IBD patients.

The most striking finding of the P16S sequence analysis was the complete lack of *Pseudomonas* species within OTU 5 in the CD patients, while OTU 5 represented 18% of species in the non-IBD patients. Sequences in OTU 5 were restricted to *Pseudomonas aeruginosa*. The loss of commensal *P.aeruginosa* in CD patients may result in dysbiosis, and could play a role in CD. *P.aeruginosa* is known to inactivate several pro-inflammatory cytokines [45]. In other settings, *P.aeruginosa* has been reported to be implicated in causing human disease, including chronic granulomatous disease in paediatric patients [46] and as a trigger in cystic fibrosis [47].

The worldwide increase in the prevalence of CD has been associated with an increased use of refrigeration [48]. There are no studies on whether changes in psychotropic bacteria (able to grow at the temperatures maintained inside a refrigerator, −1 to +10 degrees) occur in CD populations. In this study, OTU 1 that included the non-pathogenic species *P.panacis* and *P.brennerii* (reported to be present in refrigerated beef [49]) was more prevalent in CD patients than in non-IBD patients. The presence of these species in CD in relation to disease manifestation requires further investigation.

The imbalance in *Pseudomonas* species seen in our study might be a secondary consequence of defective mucosal barrier function and microbial killing present in IBD patients [50]. This could have led to an aggressive T-cell response and changed the composition of *Pseudomonas* species early in disease progression. Numerous studies have demonstrated changes in bacterial composition between beneficial and harmful bacterial species in IBD and non-IBD patients. This dysbiosis hypothesis is accompanied by a decrease in microbial diversity in active IBD [20], [21], [38], [39], [51], [52], similar what was observed with *Pseudomonas* in this study.

*Pseudomonas* is one of the most complex genera of Gram-negative bacteria with a wide range of natural habitats which might partly explain their presence in human gut. By using *Pseudomonas* 16S ribosomal analysis no single *Pseudomonas* species was identified as a causative agent of CD. However, *Pseudomonas* species were detected more frequently in CD patients and the *Pseudomonas* population in these patients was significantly less diverse when compared to that of non-IBD patients. Of particular interest was the lack of *P.aeruginosa* detected in the gut of CD patients. The dysbiosis between *Pseudomonas* species and other gut microbiota could be directly involved in CD manifestation or be a secondary consequence after establishment of disease in these children.

Finally, much of the gastrointestinal microbiota remains uncharacterised [53]. It is likely that many of the sequences detected in this study represent novel *Pseudomonas* species/strains. Full *Pseudomonas* ribosomal gene analysis (16S–23S) could provide more insights into specific *Pseudomonas* species associated with CD. Whilst the results of this study accurately represent a part of the bacteriology of active CD, this study did not address the important role that the immune system and immunological tolerance may play in CD. Specifically, it remains possible that an inappropriate immune response to commensal *Pseudomonas* species is involved in disease aetiology.

## Materials and Methods

### Patient sample collection

Patients undergoing an initial endoscopy at the Royal Children's Hospital, Melbourne, Australia for a clinical diagnosis of inflammatory bowel disease (IBD) were enrolled in this study. All of the patients had exhibited symptoms consistent with IBD. None of the patients had received antibiotics, immunosuppressives drugs or glucocorticosteroids prior to endoscopy. A total of 68 patients were included in this study, 32 children were diagnosed with Crohn's disease (CD) and 36 children were diagnosed with no pathological abnormalities (non-IBD). Mucosal biopsy specimens obtained from the ileum were used for all analyses. The presence or absence of granulomas was recorded for each CD patient. A detailed description of patient characteristics is listed in supporting tables S1 and S2, and includes presence of granulomas, inflammation, sex and age. Both patient groups had a mean age of 12 years. All tissue specimens were collected and placed immediately into a sterile screw-cap cryotube containing 0.5 ml of RNAlater [Ambion]. The samples were stored at −70°C until DNA extraction. Samples were examined in the order in which they were collected, so that CD and non-IBD cases were examined in a random manner. The clinical diagnosis of CD was established using standard clinical and endoscopic criteria according to the Vienna classification [34] and confirmed by histological examination of gut tissue. Ethics approval for the study was obtained from the Human Ethics Committee of the Royal Children's Hospital (EHRC no. 23003). Written informed consent was obtained from each individual, parent or guardian prior to enrolment in the study.

### DNA extraction from biopsy specimens

Tissue samples (3–6 mm^3^) were homogenised using a sterile plastic pestle in 200 µl of lysis buffer (20 mM Tris-HCl, pH 8.0 and 2 mM EDTA). The homogenate was subjected to three cycles of freeze/thaw. Lysozyme (20 mg/ml, Sigma) was added to each sample and incubated at 37°C for 30 minutes. A volume of 600 µl buffer AL (QIAamp DNA mini kit, Qiagen) and glass beads (∼20%, diameter 150–212 microns, Sigma) were added to each sample and subjected to mechanical disruption in a FastPrep (setting of 6.5 ms^−2^ for 45 seconds). Glass beads were removed and supernatant was treated with proteinase K (24 mAU) incubated at 56°C for 2 hours with vortexing 2–3 times per hour. DNA was extracted using the QIAamp DNA mini kit according to the manufacturer's instructions [QIAgen]. In an alternative method DNA was extracted as above except the freeze/thaw cycles and the FastPrep cycle were omitted. Briefly, tissue sample was homogenised as above in 200 µl buffer RLT [QIAgen]. Then two 5 mm sterile glass beads and an additional 400 µl buffer RLT were added and homogenate was incubated for one hour with 15 seconds vortexing every 10 minutes. Glass beads were removed and the DNA was extracted using the QIAamp DNA mini kit according to the manufacturer's instructions [QIAgen]. DNA was eluted into 50 µl nuclease free water. Two DNA extraction methods were employed for 16s rDNA amplification, since studies conducted in our lab has shown that the use of different extraction methods can influence the diversity of 16s rDNA sequences obtained (unpublished observations).

All DNA extractions were conducted in biological safety cabinet class II. A mock homogenisation and extraction without sample material was included in each extraction run to exclude contamination with bacteria during the procedure.

### Amplification of *Pseudomonas* 16S ribosomal DNA

A *Pseudomonas* specific 16S ribosomal (P16S) PCR (617 bp) was conducted using *Pseudomonas* specific primers described by Spilker *et al.*, [35]. Five µl of purified DNA was used in a 50 µl PCR reaction. PCR reactions were performed using a final concentration of 1× PCR Buffer, 2 mM MgCl, 2.5 U *Taq* polymerase [Applied Biosystems], 250 µM of each deoxynucleoside triphosphate [Invitrogen] and 500 nM of the *Pseudomonas* genus specific primers, PA-GS-F (5′-GACGGGTGAGTAATGCCTA3-′), and PA-GS-R (5′-CACTGGTGTTCCTTCCTATA-3′) [35]. PCR conditions were: 95°C for 5 minutes, 10 cycles of 94°C for 15 seconds, 53°C for 30 seconds and 72°C for 45 seconds, this was repeated for another 25 cycles with the exception that the 72°C elongation step was increased by 5 seconds every cycle. A final extension phase of 72°C for 10 minutes was used. A nested PCR assay was conducted using all DNA samples which were negative or weakly positive (not sufficient for gel extraction and cloning). Universal bacterial 16s primers described by Wang *et al.*, [36] were used in the first round PCR. PCR reactions were performed in a total volume of 50 µl using a final concentration of 1× PCR Buffer, 1.5 mM MgCl, 2.5 U Taq polymerase [Applied Biosystems], 200 µM of each deoxynucleoside triphosphate [Invitrogen] and 1 µM of universal bacterial primers, 27f (5′-AGAGTTTGATCMTGGCTCAG-3′) and 1492r (5′-TACGGYTACCTTGTTACGACTT-3′). PCR conditions were: 95°C for 5 minutes, 40 cycles of 94°C for 30 seconds, 62.5°C for 30 seconds and 72°C for 1 minute. A final extension phase of 72°C for 10 minutes was used. For the second round PCR, 5 µl of the first round 16s PCR product was used with *Pseudomonas* specific primers (PA-GS-F and PA-GS-R) in a Master Amp PreMix B buffer [EpiCentre]. PCR conditions were as above except an annealing temperature of 57°C was used. *E.coli* DNA was used as a positive control for the first round and as negative control for the second PCR. *P.aeruginosa* and *P.putida* DNA was used as a positive control for both rounds of PCR. In all PCR assays a mock extraction and a negative PCR reaction were included. PCR products were separated by electrophoresis using a 1% TBE agarose gel and visualized under UV light using ethidium bromide. Amplicons of the correct size were extracted from the agarose gel and purified using a QIAquick Gel Extraction kit [QIAGEN], according to the manufacturer's instructions.

### Cloning and sequencing of *Pseudomonas* 16S rDNA fragments

To evaluate the sequence diversity within the *Pseudomonas* species, the P16S rDNA PCR products CD patients and non-IBD patients were cloned and sequenced. DNA was extracted using the QIAquick gel extraction kit [QIAgen] and cloned into pCR2.1 vectors [TA Cloning Kit, Invitrogen] and transformed into One Shot Top 10 chemically competent *E.coli* [TA Cloning Kit, Invitrogen], as described by the manufacturer's instruction. Blue/white screening was performed and white colonies were replated for insert confirmation. A minimum of 24 white colonies from each PCR product were selected and grown overnight at 37°C in 96 well agar plates. Plates were then sent to the Australian Genome Research Facility [AGRF, Brisbane, Australia] for culture, plasmid isolation and bi-directional sequencing using the vector-targeted M13 forward and reverse primers. Sequencing reactions were performed with Big Dye version 3.1 on an AB3730x1 sequencing platform. All sequences were analysed using the Sequencher program, (version 4.7) to remove low quality data and vector sequences, and used to prepare consensus sequences of each clone.

### Bioinformatics analysis

Consensus sequences were aligned using ClustalW, available from GenomeNet Computation service from Kyoto University Bioinformatics Center (http://align.genome.jp). A PHYLIP generated DNA distance matrix (dnadist) with Jukes-Cantor option was calculated using the Mobyle Pasteur program suite (http://mobyle.pasteur.fr). The PHYLIP dnadist matrix was used for LIBSHUFF gene library comparison and for the generation of operational taxonomic units using DOTUR. The LIBSHUFF analysis compares two gene libraries and provides a statistical test for the null hypothesis to determine if two libraries are significantly different from one another [32]. In this study webLIBSHUFF version 0.96 was used (Henrikson, JR. 2004 http://libshuff.mib.uga.edu). The DOTUR program (http://www.plantpath.wisc.edu/fac/joh/dotur.html) [33] was used to assess the richness of *Pseudomonas* collection associated with biopsy specimens obtained from CD and non-IBD patients. In our study we compared the *Pseudomonas* membership (the list of OTUs in a community) and the membership structure (the abundance of each OTU) at a 97% sequence similarity threshold. The number of sequences in each unique OTU and the proportion of sequences from each patient group in each unique OTU was calculated and analysed.

### Phylogenetic analysis

Phylogenetic analyses was conducted using MEGA version 3.1 (Kumar, Tamura, Nei 2004) [37]. Consensus sequences from each individual OTU group were generated using Sequencher program (version 4.7) and sequences for 85 *Pseudomonas* reference strains for use in the phylogenetic tree were obtained from the Ribosomal Database Project II (release 9.59 at http://rdp.cme.msu.edu). All sequences were adjusted to 617 bp and OTUs consensus sequences were aligned with *Pseudomonas* type strains using ClustalW from MEGA then a phylogenetic tree was constructed. Trees were produced by the neighbor-joining program with the Kimura correction and Boot strapping 500 times.

### Statistical analysis

Fisher's exact test was used to analyse the association between two binary variables, such as the presence or absence of *Pseudomonas* between CD and non-IBD patient groups. Tests were either one or two tailed and a p value of <0.05 was considered significant.

## Supporting Information

Table S1 Characteristics of CD and non-IBD control patients
(0.13 MB DOC)

Table S2 Summary of patient characteristics
(0.03 MB DOC)

Table S3 Summary of OTUs in Pseudomonas 16S gene libraries.
(0.04 MB DOC)
