# Supplementary material for: Identification and Characterisation of Pseudomonas 16S Ribosomal DNA from Ileal Biopsies of Children with Crohn's Disease
Source: PLoS One. 2008 Oct 31;3(10):e3578. doi: 10.1371/journal.pone.0003578 (PMC2572839; doi:10.1371/journal.pone.0003578)
Supplement: Table S1 — Characteristics of CD and non-IBD control patients (0.13 MB DOC) [file pone.0003578.s001.doc]

**Table S1.** Characteristics of CD patients and Non-IBD Control Patients

| **Patient number** | **Age (yrs)** | **Sex** | **Granuloma present** | **Visible inflammation** | **Pseudomonas 16s DNA+** | |
| --- | --- | --- | --- | --- | --- | --- |
| CD1 | 14.7 | M | No | Yes | - |  |
| CD2 | 12 | M | No | Yes | + |  |
| CD3 | 10.9 | M | Yes | Yes | - |  |
| CD4 | 11.5 | M | No | Yes | + | Sequenced |
| CD5 | 17.6 | F | No | Yes | + | Sequenced |
| CD6 | 13 | F | No | No | - |  |
| CD7 | 11.9 | F | No | Yes | + | Sequenced |
| CD8 | 15.4 | M | Yes | Yes | + |  |
| CD9 | 17 | F | No | Yes | + | Sequenced |
| CD10 | 9.1 | M | Yes | No | - |  |
| CD11 | 14.8 | f | Yes | No | + | Sequenced |
| CD12 | 9.4 | m | Yes | Yes | - |  |
| CD13 | 12.8 | f | Yes | Yes | + | Sequenced |
| CD14 | 10.5 | m | Yes | Yes | - |  |
| CD15 | 13.3 | m | Yes | Yes | - |  |
| CD16 | 10.3 | f | Yes | Yes | + | Sequenced |
| CD17 | 6.5 | m | No | Yes | + | Sequenced |
| CD18 | 14.4 | m | Yes | Yes | + | Sequenced |
| CD19 | 13.4 | m | Yes | Yes | - |  |
| CD20 | 10.1 | f | No | No | + | Sequenced |
| CD21 | 8.1 | m | Yes | No | - |  |
| CD22 | 5.4 | m | Yes | No | - |  |
| CD23 | 14.5 | m | Yes | Yes | + | Sequenced |
| CD24 | 12.2 | m | No | No | - |  |
| CD25 | 11.5 | m | Yes | No | + |  |
| CD26 | 12.6 | m | No | Yes | + | Sequenced |
| CD27 | 7.2 | f | No | No | - |  |
| CD28 | 11.1 | m | No | Yes | + |  |
| CD29 | 13.8 | m | Yes | No | - |  |
| CD30 | 13.3 | f | No | Yes | + | Sequenced |
| CD31 | 11.2 | f | Yes | Yes | - |  |
| CD32 | 13.8 | f | Yes | No | + |  |
| NO1 | 11.9 | f | No | No | - |  |
| NO2 | 13.4 | m | No | No | - |  |
| NO3 | 16.8 | f | No | No | + | Sequenced |
| NO4 | 11.2 | f | No | No | + | Sequenced |
| NO5 | 16.1 | f | No | No | + | Sequenced |
| NO6 | 14.5 | m | No | No | - |  |
| NO7 | 10.8 | f | No | No | + | Sequenced |
| NO8 | 15.6 | m | No | No | - |  |
| NO9 | 8 | m | No | No | - |  |
| NO10 | 10.5 | m | No | No | - |  |
| NO11 | 16.9 | f | No | No | + | Sequenced |
| NO12 | 12.2 | m | No | No | - |  |
| NO13 | 14.8 | f | No | No | - |  |
| NO14 | 11.2 | m | No | No | - |  |
| NO15 | 9.6 | m | No | No | + | Sequenced |
| NO16 | 16.5 | f | No | No | + | Sequenced |
| NO17 | 5 | f | No | No | - |  |
| NO18 | 10.7 | m | No | No | - |  |
| NO19 | 9.5 | f | No | No | - |  |
| NO20 | 17.8 | f | No | No | + | Sequenced |
| NO21 | 17.2 | m | No | No | - |  |
| NO22 | 11.4 | f | No | No | - |  |
| NO23 | 14.3 | f | No | No | + | Sequenced |
| NO24 | 6.9 | f | No | No | - |  |
| NO25 | 18.2 | f | No | No | - |  |
| NO26 | 6.5 | f | No | No | + | Sequenced |
| NO27 | 11 | f | No | No | - |  |
| NO28 | 4.1 | m | No | No | + | Sequenced |
| NO29 | 14.1 | f | No | No | - |  |
| NO30 | 7.4 | m | No | No | - |  |
| NO31 | 3.2 | f | No | No | + | Sequenced |
| NO32 | 13.3 | f | No | No | - |  |
| NO33 | 11.4 | f | No | No | - |  |
| NO34 | 13.8 | m | No | No | - |  |
| NO35 | 12.4 | m | No | No | - |  |
| NO36 | 14.6 | f | No | No | - |  |

Thirty two Crohn’s disease patients (CD) and 36 Non-inflammatory bowel disease patients (NO) were analysed in the study. The age and sex of each patient were recorded. The presence of granulomas in ileal tissues was analysed by histological examination. Visible inflammation at the collection side was also recorded during endoscopy. Patients which were positive (+) for *Pseudomonas* 16S rDNA are shown, and *Psedumonas* 16S rDNA fragments which were sequenced are indicated.
