# Supplementary material for: Identification and Characterisation of Pseudomonas 16S Ribosomal DNA from Ileal Biopsies of Children with Crohn's Disease
Source: PLoS One. 2008 Oct 31;3(10):e3578. doi: 10.1371/journal.pone.0003578 (PMC2572839; doi:10.1371/journal.pone.0003578)
Supplement: Table S2 — Summary of patient characteristics (0.03 MB DOC) [file pone.0003578.s002.doc]

**Table S2.** Summary of Patient Characteristics

| **Patient characteristics** | **CD** | **Non-IBD** |
| --- | --- | --- |
| **Patient number** | 32 | **36** |
| **Sex ratio (male:female)** | 21:11 | **14:22** |
| ***Pseudomonas* 16S positive patients** | 58% (18/32) | **33%* (12/36)** |
| **Male : Female ratio** | 9:9 | **3:9** |
| **Age (average in years)** | 12.0 | **12.0** |
| **Age range** | **5.4-17.6** | **3.2-18.2** |

Abbreviations: CD = Crohn’s disease, non-IBD = non inflammatory bowel disease, * significant difference versus CD group using Fisher exact test (p<.0.05)
