# Supplementary material for: Identification and Characterisation of Pseudomonas 16S Ribosomal DNA from Ileal Biopsies of Children with Crohn's Disease
Source: PLoS One. 2008 Oct 31;3(10):e3578. doi: 10.1371/journal.pone.0003578 (PMC2572839; doi:10.1371/journal.pone.0003578)
Supplement: Table S3 — Summary of OTUs in Pseudomonas 16S gene libraries. (0.04 MB DOC) [file pone.0003578.s003.doc]

**Table S3.** Summary of OTUs in *Pseudomonas* 16s gene libraries

|  | | **CD** | **Non-IBD** | **Total** |
| --- | --- | --- | --- | --- |
| **Number of patients** | | 13 | 12 | 25 |
| **Number of sequences** | | 320 | 261 | 581 |
| **Number of unique OTUs (Sequence similarity threshold 97%)** | | 5 | 6 | 6 |
| **Number of sequences in each OTU** | OTU 1 | 215 | 113 | 328 |
|  | OTU 2 | 60 | 34 | 94 |
|  | OTU 3 | 36 | 22 | 58 |
|  | OTU 4 | 2 | 26 | 28 |
|  | OTU 5 | 0 | 47 | 47 |
|  | OTU 6 | 7 | 19 | 26 |

Abbreviations: CD = Crohn’s disease, Non-IBD = Non inflammatory bowel disease, OTU = Operational taxonomic unit
